# Evaluation of Risk Factors and a Gene Panel as a Tool for Unexplained Infertility Diagnosis by Next-Generation Sequencing

**DOI:** 10.3390/medicina61020271

**Published:** 2025-02-05

**Authors:** Eglė Jašinskienė, Ieva Sniečkutė, Ignas Galminas, Lukas Žemaitis, Mantas Simutis, Marija Čaplinskienė

**Affiliations:** 1 Department of Biochemistry, Vytautas Magnus University, K. Donelaicio St. 58, 44248 Kaunas, Lithuania; m.caplinskiene@gmail.com; 2 UAB Genomika, K. Barsausko St. 59, 51423 Kaunas, Lithuania; ievasniec@gmail.com (I.S.); ignas@genomika.lt (I.G.); lukas@genomika.lt (L.Ž.); info@genomika.lt (M.S.)

**Keywords:** unexplained infertility, NGS diagnosis, genetic testing, sociodemographic factors, gene panel, risk factors, genetic factors, evaluation, next-generation sequencing (NGS) technology

## Abstract

*Background and Objective:* Unexplained infertility is a major challenge in reproductive medicine and requires advanced diagnostic approaches to identify the underlying factors accurately. This study aims to evaluate the utility of risk factor analysis and a gene panel in diagnosing unexplained infertility using the next-generation sequencing (NGS) technology. Our study aimed to characterize and identify risk and genetic factors associated with unexplained infertility. *Materials and methods:* A cohort of patients with unexplained infertility was comprehensively screened for risk factors and genetic variations using a targeted gene panel (10 couples with unexplained infertility (UI) and 36 fertile couples). 108 articles were selected (58 on female infertility and 50 on male infertility) presenting genes that may be associated with unexplained infertility. A gene panel for unexplained infertility was compiled based on the literature data. A customized virtual panel was created from the exome sequencing data. *Results:* In the female group, controls had a higher mean age, while in the male patients, both groups were similar in terms of age. Both gender groups had comparable BMI values. No significant associations (*p* > 0.05) between risk factors and unexplained infertility were found when evaluating anthropometric parameters and other sociodemographic characteristics. In two male patients (20%), a molecular defect was detected in NGS variants classified aspossible benign and probably benign In particular, missense variants were identified in the *UGT2B7* and *CATSPER2* genes, A molecular defect classified as probably damaging was found in five female patients (50%). In particular, missense variants were identified in the *CAPN10*, *MLH3*, *HABP2*, *IRS1*, *GDF9*, and *SLC19A1* genes. *Conclusions:* The study emphasizes that unexplained infertility is often related to mechanisms beyond causative mutations and highlights the need for integrative genomic research involving broader gene panels and multi-faceted approaches, including transcriptomics and epigenetics, to uncover latent genetic predispositions.

## 1. Introduction

Infertility is a condition that not only causes physical disability but also exerts a significant social and psychological impact, as couples are unable to fulfill one of their most fundamental desires: becoming parents. As such, infertility represents a pressing issue with profound implications, not only for the affected couple but also for social and demographic indicators.

Infertility is a heterogeneous pathology caused by a variety of factors, including environmental and genetic influences. It is estimated that nearly 50% of infertility cases can be attributed to genetic defects. However, the exact cause of infertility often remains elusive, as identifying genetic factors in clinical practice can be particularly challenging. With the sequencing of the human genome and the increasing shift toward personalized testing and treatment in medicine, new opportunities have emerged to use next-generation sequencing (NGS) technologies to investigate the reproductive system. Currently, significant attention is focused on studying the genetic factors that influence the reproductive system and infertility, as well as exploring potential treatment strategies. As a result, there is a growing body of literature documenting the impact of genetic factors on infertility. Numerous studies and systematic reviews have identified target genes that play a role in infertility. Researchers suggest that as more studies are conducted, additional genes influencing infertility will likely be discovered. Understanding the etiology of infertility is essential for elucidating the pathophysiology of the human reproductive system, establishing accurate diagnoses and applying personalized treatments. One such study has proposed a gene panel for infertility that can be assessed using NGS. A total of 87 genes have been identified as influencing male and female infertility. This NGS gene panel was analytically validated through a retrospective analysis of 118 genomic DNA samples, known to be significant for infertility. The results demonstrated an accuracy of over 99%, with 98% sensitivity for single nucleotide variants and 91% sensitivity for deletions [1]. Furthermore, the European Society of Human Genetics (ESHG) and the European Society of Human Reproduction and Embryology (ESHRE) have presented data at one of their congresses regarding genes confirmed to influence male and female infertility [2].

The constantly changing environmental and lifestyle conditions affecting the human body on a daily basis significantly influence fertility. Modern life changes are driven by a variety of factors, including industrial development, exposure to chemicals and toxins, and unfavorable lifestyle habits, all of which impact the body, including the reproductive system. Factors such as age, gender, education, body mass index (BMI), sauna, fast food, preservatives in food, radiation, physical activity, pesticides, smoking, alcohol consumption, and stress levels play an important role in understanding the social patterns and risk factors associated with unexplained infertility [3]. Many authors suggest that these risk factors have a more pronounced impact on the reproductive system in cases of diagnosed infertility compared to the general population.

Looking at the literature, not all basic tests (biochemical markers, instrumental tests, and semen analysis) help to identify the exact cause of infertility and to select an individual treatment. This is particularly important in the diagnosis of unexplained infertility. In the case of unexplained infertility, a thorough examination is essential, as the causes may be immunological, endocrinological, or genetic in origin. Therefore, the selection of target genes and the development of a gene panel for the investigation of unexplained infertility, using the latest technologies and tools, will ensure high-quality diagnostic results and contribute to the solution of infertility problems.

The study aims to improve the diagnostic capabilities of male and female patients with infertility by using an NGS panel to identify genetic variants and statistically significant sociodemographic factors that can provide valuable insights for a more personalized and accurate management of unexplained infertility.

## 2. Materials and Methods

The study was divided into two parts. The first part focused on the creation, adaptation, and clinical application of gene panels for unexplained infertility. To achieve this, a systematic literature review was conducted in adherence to the PRISMA (Preferred Reporting Items for Systematic Review and Meta-Analyses) protocol. Clinical studies were searched and analyzed using thePubMed, MEDLINE, SCOPUS, and Cochrane databases, with a focus on publications from 2010 to 2020. The search utilized the following keywords: unexplained infertility, NGS diagnosis, genetic testing, sociodemographic factors, gene panel, risk factors, genetic factors, evaluation, and Next-Generation Sequencing (NGS) technology. The database searches were refined using the following filters: 10 years, humans, female, and male. Upon entering the keyword combinations into the databases, 2880 bibliographic records were retrieved. In the first phase, studies conducted more than 10 years ago and those involving non-human subjects were excluded using the aforementioned filters. In the second phase, the titles and abstracts of all identified articles were reviewed, and publications potentially meeting the inclusion criteria were shortlisted based on abstract analysis. In the third phase, the full texts of the shortlisted articles were thoroughly reviewed, with a specific focus on the genes identified in the studies and their relevance to unexplained infertility. Based on the inclusion and exclusion criteria, 105 articles were selected for the study: 58 related to female infertility and 47 to male infertility [4,5,6,7,8,9,10,11,12,13,14,15,16,17,18,19,20,21,22,23,24,25,26,27,28,29,30,31,32,33,34,35,36,37,38,39,40,41,42,43,44,45,46,47,48,49,50,51,52,53,54,55,56,57,58,59,60,61,62,63,64,65,66,67,68,69,70,71,72,73,74,75,76,77,78,79,80,81,82,83,84,85,86,87,88,89,90,91,92,93,94,95,96,97,98].

The second part of the study explored the relationships between genetic markers, risk factors, and unexplained infertility in couples diagnosed with this condition. A case–control design was utilized to evaluate a smaller sample of patients and control subjects, aiming to identify potential associations between risk factors and unexplained infertility. This approach was also selected due to the costliness of genetic analysis and ethical considerations related to the investigation of genetic information. Each couple was analyzed as an individual clinical case, with evaluations covering medical history, risk factors, biochemical markers, treatments applied, and their outcomes. While the small number of participants may limit the generalizability of the findings, this is a preliminary study aimed at gathering evidence on the impact of unexplained infertility. In the future, it could serve as a foundation for larger studies (such as cohort studies or randomized clinical trials), providing more robust evidence regarding potential cause-and-effect relationships.

The study was conducted between 2022 and 2024, with research samples collected at the Lithuanian University of Health Sciences Kaunas Hospital and the Fertility Clinic. This reproductive center was specifically chosen for its long-standing operation since 2000 and its status as one of the largest facilities in Lithuania addressing infertility issues. It is also the leading institution in the country for performing assisted reproductive procedures (446 per year).

The study group comprised 10 couples (10 women and 10 men) diagnosed with unexplained infertility. The control group consisted of 34 couples (34 women and 34 men), in which risk factors were evaluated. The age of the participants (both women and men) ranged from 19 to 42 years.

By random selection, 10 couples seeking infertility treatment for the first time at the Fertility Clinic were included in the study group. They were diagnosed with infertility following the WHO recommendations, after ruling out other potential causes: ovulatory disorders; insufficient ovarian reserve (evaluated through hormonal tests for FSH, LH, E2, TTH, T, SHBG, and AMH on day 3 of the menstrual cycle and progesterone and prolactin on day 21 of the menstrual cycle); tubal damage (diagnostic laparoscopy with chromosalpingoscopy); uterine and peritoneal damage (ultrasound examination of the internal genitalia and diagnostic laparoscopy); and male-related causes (hormonal tests for FSH, LH, T, semen analysis, and consultation with a urologist/andrologist). The couples consented to participate in the study.

The control group comprised fertile couples, each with at least one healthy child. Patients who had previously undergone abdominal surgery or infertility treatments were excluded. Subjects with conditions such as hypertension, diabetes, endocrine disorders, autoimmune or immunological diseases, other chronic illnesses, or genetic disorders were also excluded. Additionally, participants who declined to sign the consent form or withdrew from the study were excluded.

Genomic analysis: Genomic analysis was performed using next-generation sequencing (NGS) on DNA samples from patients with unexplained infertility. The workflow was performed according to standard protocols in an accredited laboratory (CAP, CLIA and DIN EN ISO 15189:2014), CeGaT GmbH (Tübingen, Germany), and included high-throughput sequencing on an Illumina NovaSeq 6000 platform. The following steps outline the methods used in this study for sample preparation, sequencing, bioinformatic processing, and variant analysis.

Sample preparation and quality control: Each sample was collected on FTA cards (Flinders Technology Associates (From CeGaT GmbH laboratory, Tübingen, Germany)) and underwent an initial quality assessment by fluorescence-based quantification to ensure that DNA quantities met the minimum threshold for further processing (average DNA concentration in all samples: 9.6–18 ng/µL). Approximately 50 ng of DNA per sample was used to generate sequencing libraries using the Twist Human Core Exome + RefSeq + Mitochondrial panel.

Sequencing parameters: Sequencing was performed using a 2 × 100 bp paired-end configuration, achieving a Q30 score of 94.31%, indicating high quality sequencing data. The sequencing depth and number of reads varied from sample to sample, ranging from 63 to 180 million reads per sample, ensuring sufficient coverage of target genomic regions (average coverage: 99.4% for most samples).

Bioinformatic processing: After sequencing, reads were demultiplexed using Illumina (San Diego, CA, USA) bcl2fastq software (v2.20), followed by adapter trimming with Skewer (v0.2.2). Reads were aligned to the hg19 reference genome using the Burrows–Wheeler Aligner (BWA-mem, v0.7.17-cegat), ensuring high alignment accuracy. Pseudoautosomal regions on chromosome Y were masked to avoid mapping errors across chromosomes. After alignment, ABRA (v2.18) was used to perform a local realignment, allowing precise detection of insertions and deletions (indels).

Variant detection and filtering: The variants were identified using algorithms developed by CeGaT GmbH (DE). Variants included single nucleotide variants (SNVs), indels, and copy number variations (CNVs), which were filtered to focus on genes associated with unexplained infertility. Coverage comparisons with a reference cohort were used to identify CNVs, while annotation was performed using public databases such as ClinVar and dbSNP to determine clinical significance. Low frequency alleles were included in the analysis, with the threshold for observed frequency being as low as 2%.

Variant annotation and interpretation: Variants were annotated and categorized by pathogenicity according to the guidelines of the American College of Medical Genetics and Genomics (ACMG), with a focus on likely pathogenic as well as pathogenic and uncertain significance variants for further analysis. In addition to literature sources, public databases such as ClinVar, dbSNP, and gnomAD were used to assess pathogenicity and clinical relevance. Tools such as PolyPhen-2 and SIFT assessed the potential impact of each variant on protein structure and function. To focus on rare variants potentially associated with unexplained infertility, variants commonly found in general population databases were excluded.

### Research and Diagnostic Procedure Workflow

In addition, we used an NGS gene panel in a case group (10 male and 10 female patients) with UI. The panel consisted of 29 genes for male patients and 75 for female patients to investigate the genetic causes of unexplained infertility.

The demographic and risk factors were collected anonymously through the questionnaire, which included the following information: subject code, completion date, date of birth, gender, education level, and collected medical history regarding risk factors. The questionnaire was constructed based on existing literature, focusing on the most relevant risk factors for fertility (see Table 1).

Data management and statistical analysis plan. The association between the categorical variables in the study population was tested using the chi-square test at the statistically significant level of *p* = 0.05. The reports were examined and statistically analyzed using SPSS 29.0 software. SPSS 29.0 for Windows (Statistical Package for Social Sciences) and Microsoft 365^®^ computer software (IBM SPSS Statistics 29, New York 10504-1722, NY, USA).

## 3. Results

### 3.1. Gene Panel Characteristics

The selection of target genes for unexplained infertility led to the development of a set of genetic markers applicable in clinical practice. A list of these genes can be found in Table 2.

A customized next-generation sequencing (NGS) panel was used to analyze 10 pairs (10 female and 10 male patients) with unexplained infertility. The results of the study showed that the distribution of genetic variants in the cohort of women who underwent genetic alteration screening was examined, revealing 75 genetic alterations. The total number of genetic variants ranged from 202 to 329 per sample, with an average of approximately 279 variants per sample. The study examined synonymous, missense, and nonsense genetic alterations, with synonymous accounting for 30.2%, missense for 24.7%, and nonsense for 0.001%. The distribution of genetic variants in the cohort of men who underwent genetic alteration screening was examined, revealing 29 genetic alterations. The total number of genetic variants ranged from 37 to 88 per sample, with an average of approximately 73 variants per sample. The study examined synonymous, missense, and nonsense genetic alterations, with synonymous accounting for 23.3%, missense for 28.3%, and nonsense for 0.001%.

The genetic variants identified by NGS in the studied population are shown in Table 3 for male patients and in Table 4 for female patients.

NGS of benign and probably benign variants revealed a molecular defect in two male patients (20%). Missense variants were identified in the genes *UGT2B7* and *CATSPER2*; a molecular defect was detected in 5 female patients (50%). Specifically, missense variants were identified in the genes *CAPN10*, *MLH3*, *HABP2*, *IRS1*, *GDF9*, and *SLC19A1*.

### 3.2. Anthropometry and Sociodemographic Characteristics of Study Participants

The overall response rate in the current study was 10 (100%) for cases and 34 (100%) for controls.

In total, the study comprised 44 participants, including 10 women and 10 men with UI as cases, and 34 fertile women and 34 fertile men as controls. The participants were divided into two groups according to gender.

In the female group, the control subjects had a higher average age, while in the male patients, both groups were similar in terms of age. Both gender groups had comparable BMI values.

The mean age of the female cases was 31.72 years (SD 5.22, range: 27–37), while the controls had a mean age of 28.65 years (SD 5.55, range: 20–39). The mean BMI values were 24.67 (SD 4.08) for female cases and 24.41 (SD 4.38) for controls.

In addition to the anthropometric parameters mentioned above, other sociodemographic characteristics of the study participants such as education, sauna, fast food, preservatives in food, laptop position, microwave, anabolic steroids, drugs, physical activity, pesticides, smoking, alcohol consumption, and stress levels were examined.

There was no statistically significant difference between the two study groups: female patients in Table 5 and male patients in Table 6.

Effect size estimation (Cramer’s V) indicated a small association between unexplained female infertility and age.

However, the clinical history variables showed no significant association (*p* > 0.05) with unexplained female infertility and were reported in the group as follows: The majority of study participants almost never go to the sauna (100% in cases vs. 94.2% in controls), rarely eat fast food (60% in cases vs. 32.4% of the controls), consume preservatives in food only a few times a week (60% of the cases vs. 47.1% of the controls), put the laptop on the table (50% of the cases vs. 88.2% of the controls), never take anabolic steroids and pesticides, most do not take drugs (60% of the cases vs. 94.2% of the controls), and the majority do not smoke (50% of the cases and 91.2% of the controls). Stress levels were also almost the same in both groups, with the second stress level predicted in 80% of cases and 88.2% of controls.

In addition, the three physical activity levels (sedentary (a few times per month and rarely), moderately active (a few times per week), and very active (daily)) were the same in both groups, and the majority of study participants had a sedentary lifestyle (70% in cases versus 61.8% in controls).

A higher educational status characterized the case group: the majority of the case group had a university degree (80%), while most of the control group had a higher education degree (29.4%).

The average age of the male cases was 32.52 years (SD 4.55, range: 28–37), while the control group had an average age of 31.32 years (SD 5.54, range: 22–40). The average BMI values were 26.37 (SD 4.44) for the cases and 27.54 (SD 4.53) for the controls.

In addition, there were no significant associations (*p* > 0.05) between medical history variables and unexplained female infertility. The characteristics reported in both groups were similar: Most participants rarely visited the sauna (100% for cases vs. 94.2% for controls), rarely consumed fast food (60% for cases vs. 32.4% for controls), had a limited intake of preservatives (60% for cases vs. 47.1% of controls), put laptops on the table (50% of cases vs. 88.2% of controls), did not use anabolic steroids or pesticides, most did not take medication (60% of cases vs. 94.2% of controls), and were non-smokers (50% of cases vs. 91.2% of controls). Stress levels were comparable between the two groups, with an 80% prediction of a second stress level for cases and 88.2% for controls.

Levels of physical activity, categorized as sedentary (infrequent and infrequent activity), moderately active (a few times per week), and very active (daily), were also similar in both groups, with the majority leading a sedentary lifestyle (70% for cases versus 61.8% for controls). Notably, a higher proportion of people in the case group had a university degree (80%) than in the control group, where the majority had a lower level of education (29.4%).

## 4. Discussion

Infertility is a complex disorder that affects about 10–15% of couples worldwide. In 10–25% of cases, the cause of infertility remains unclear even after a comprehensive clinical examination. Next-generation sequencing (NGS) technologies offer a unique opportunity to better understand the genetic basis of infertility, especially in cases where conventional diagnostic methods prove ineffective. These technologies not only expand diagnostic capabilities but also have the potential to enable new scientific discoveries that can refine existing infertility treatment and prevention strategies.

This study highlights the rare presence of causative gene variants in unexplained infertility and supports the assumption that germline mutations directly attributable to infertility are relatively rare. The results suggest that while single-gene variants may contribute to reproductive problems, unexplained infertility is likely due to a multifactorial etiology involving subtle genetic interactions rather than single, identifiable mutations. The limited discovery of clinically actionable variants in this cohort suggests that unidentified complex genetic or polygenic factors, in addition to environmental influences, may play a critical role in unexplained infertility.

The use of NGS expands the diagnostic scope beyond conventional methods. However, the study emphasizes that unexplained infertility is often related to mechanisms that go beyond the causative mutations. This underscores the need for integrative genomic research that includes broader gene panels and multi-faceted approaches, including transcriptomics and epigenetics, to uncover hidden genetic predispositions [99].

### Recommendations for Further Research

Expand genomic and polygenic research: Future studies should include an expanded range of genes in NGS panels to capture possible polygenic contributions to infertility. This approach would facilitate the identification of gene–gene interactions that may subtly influence reproductive outcomes, thus improving diagnostic accuracy in idiopathic infertility.

Include epigenomic and transcriptomic profiles: Given the infrequent identification of germline mutations, a focus on additional genomic layers—such as epigenetic modifications and RNA expression profiles—could provide insights into the regulatory mechanisms of infertility. Examining methylation patterns, histone modifications, and transcriptomic data in infertility patients could uncover previously overlooked contributing factors.

Establish uniform standards for the use of NGS in infertility: The development of standardized guidelines for NGS diagnostics in infertility would allow for more consistent variant analysis and cross-study comparability, which would improve both data interpretation and the establishment of robust genotype–phenotype correlations in the field of reproductive health.

Emphasize longitudinal studies: To validate and refine genomic findings, longitudinal studies investigating infertility in different populations and under different environmental conditions are essential. Such studies could reveal region-specific genetic variants and gene–environment interactions, thus improving the generalizability and clinical relevance of genetic infertility research.

By implementing these research guidelines, the field can make progress in understanding unexplained infertility and promote a shift from single-gene studies to comprehensive genomic analyses that consider the complex interplay of genetic, epigenetic, and environmental factors. This integrative approach will support the development of precision diagnostics and personalized treatment options in reproductive medicine.

This study, together with other scientific sources, shows that risk factors related to lifestyle, sociodemographics, and other factors can influence infertility. However, these effects are often multifactorial and are not always directly related to infertility of an unknown origin. The lifestyle indicators examined in this study, including consumption of fast food, food preservatives, sauna visits, physical activity, and smoking showed no significant differences between the study participants and the control group. However, the scientific literature emphasizes that even minor lifestyle changes can have an impact on reproductive health. For example, studies show that the frequent consumption of fast food and food preservatives can affect hormone balance and spermatogenesis or egg quality through oxidative stress and inflammatory processes [100,101,102].

In the present study, a lack of physical activity was found in both study groups, with the prevalence of this problem being 70% in the study group and 61.8% in the control group. This result indicates that a sedentary lifestyle is a significant risk factor for infertility. A sedentary lifestyle is associated with obesity and metabolic disorders, which can affect reproductive function. Regular physical activity has been shown to reduce oxidative stress and improve hormone balance, both of which are crucial factors for fertility.

Education level is also an important factor. In this study, a higher percentage of participants in the study group had a university degree (80%) than in the control group (29.4%). The literature suggests that a higher level of education is often associated with greater lifestyle awareness and better access to healthcare. However, the present study also shows that a higher level of education may be associated with later pregnancy planning, which in turn increases the risk of infertility due to the effects of age.

Another important aspect is the level of stress, which was found to be almost equal between the two groups in this study. Research has shown that chronic stress impairs spermatogenesis and leads to a decrease in testosterone levels, inhibition of gonadotropin secretion, and a decrease in the functionality of the reproductive organs [103,104,105]. Despite the lack of statistically significant differences in this study, it is important to emphasize the importance of stress management in the treatment of infertility.

In conclusion, the results of this study suggest that lifestyle and sociodemographic factors may have some impact on unexplained infertility, although this impact is not always significant. These results underscore the need for further studies that use integrated methods and include genetic, epigenetic, and lifestyle variables to analyze the impact of these factors in more detail.

## 5. Conclusions

A systematic review of the literature was conducted, resulting in the identification of a gene panel that may be relevant to the diagnosis and treatment of unexplained infertility. The gene sets in question include both autosomal and sex-linked genes associated with spermatogenesis, oogenesis, hormone regulation, genome stability, and cell cycle processes. The application of these gene sets in clinical practice in conjunction with advanced technologies such as next-generation sequencing (NGS) offers several advantages, including a more precise identification of genetic variants, a personalized diagnosis, a better understanding of gene–phenotype relationships, the development of new tools to identify genetic etiology, and a better understanding of infertility.

A case–control study of 10 couples (10 women and 10 men) to investigate unexplained infertility found no pathological genetic variants of clinical significance in any of the groups studied. An advanced next-generation sequencing (NGS) approach was used to identify a number of genes potentially associated with infertility of an unknown origin. Despite the sensitivity of the method and its ability to detect a wide range of genetic variations, no confirmed genetic abnormalities were found in the study group that could explain this type of infertility. To further refine the etiology of unexplained infertility, it is recommended to study a larger sample that includes a broader range of genetic and epigenetic data and also takes into account possible gene–environment interactions.

The results of this study showed that there were no statistically significant differences between the study and control groups in the assessment of risk factors. The study also found a modest but statistically significant correlation between infertility and age in women of uncertain parentage, suggesting that age may be one of several factors associated with infertility. The analysis showed that the majority of participants in both groups had a sedentary lifestyle. However, there was a difference between the study and control groups in terms of education level: A higher proportion of the study group had a university degree, while in the control group, the education level was predominantly post-secondary. In addition, there were slight differences in age and body mass index (BMI) between the study subjects and the control group. The results of this study provide valuable insight into the sociodemographic and lifestyle factors associated with infertility of an uncertain origin. However, it is important to point out that the study did not reveal any specific clinical or medical associations. Future research should focus on analyzing these factors in more detail using more comprehensive methods to better understand their impact on infertility. Compiling the questionnaire on risk factors will help reproductive physicians to assess their impact on couples’ reproductive systems and make patients aware of the possible causes of infertility.

## Figures and Tables

**Table 1:** Risk factors associated with infertility.

Harmful habits Alcohol intake Smoking
Nutrition Diet and inadequate nutrition Obesity Consumption of preservatives
Low-frequency electromagnetic fields Mobile phones Laptops Microwave ovens
Noise and psychological stress
Thermal effects
Pesticides
Heavy metals
Medications, anabolic steroids
Chlorinated water

**Table 2:** Genes associated with unexplained male and female infertility included in the custom NGS panel. UI—unexplained infertility. The description of female genes is in Appendix A. The description of male genes is in Appendix B.

Genes for Male UI	Genes for Female UI
*MTHFR*; *FOLR1*; *TCN2*; *CTH*; *C19A1*; *SYCP3*; *CYP19A1*; *ESR1*; *LIF*; *HABP2*; *MLH3; TUBB8*; *ZP1*; *PADI6*; *TLE6*; *F2*; *CFTR*; *CAPN10*; *AR*; *FSHR*; *LHCGR*; *ACE*; *PAI-1*; *SOD2*; *RNLS*; *VDR*; *PALT2*	*CATSPER1*; *POLG*; *RPL23A*; *RPL4*; *RPS27A*; *RPS3*; *RPS8*; *TOMM7*; *MTHFR*; *APLF*; *CYB5R4*; *ERCC4*; *TNRFSF21*; *MORC1*; *PIWIL1*; *ZFAND6*; *RBMY1F*; *DPY19L2*; *ADAM3A*; *NXF2*; *SIRPB1*; *FSHR*; *LHCGR*; *AR*

**Table 3:** Variants identified in the male patients with unexplained infertility.

Case	Gene (Transcript Isoform)	SNP ID	Polyphen	Consequence	Clinical Relevance
1	*UGT2B7*	rs61361928 COSV100535388 COSV5944185	Probably damaging	Missense variant	Likely benign, Drug response
2	*CATSPER2*	rs11638719	Possibly damaging	Missense variant, Transcript variant	Not reported in ClinVar

**Table 4:** Variants identified in the female patients with unexplained infertility.

Case	Gene (Transcript Isoform)	SNP ID	Polyphen	Consequence	Clinical Relevance
1	*CAPN10*	rs201157354	Probably damaging	Missense variant	Likely benign
2	*MLH3*	rs28756986 CM013005 COSV53156471	Probably damaging	Missense variant	Benign/likely benign
3	*HABP2*	rs7080536 CM032937 COSV63490531	Probably damaging	Missense variant	Risk factor/benign
4	*GDF9*	rs61754582 CM066833	Probably damaging	Missense variant	Likely benign
5	*SLC19A1*	rs578206452 COSV60589312	Probably damaging	Missense variant	Likely benign
6	*IRS1*	rs41265094 CM942133 COSV99043455	Probably damaging	Missense variant	Likely benign

**Table 5:** Risk factors and their association with female cases: *p*-values analysis.

Variable	Categories	Cases (n = 10), N%	Control (n = 34), N%	*p* Value
Age group	18–30 31–35 36–40 >41	2 (20) 7 (70) 1 (10) 0 (0)	20 (58.8) 10 (29.4) 4 (11.8) 0 (0)	0.582
BMI (kg/m^2^) ^1^	<18.5 18.5–25 25–30 >30	0 (0) 9 (90) 1 (10) 0 (0)	1 (2.9) 21 (61.8) 10 (29.4) 2 (5.9)	0.574
Education	Primary High school Higher school University University degree	0 (0) 2 (20) 0 (0) 0 (0) 8 (80)	2 (5.9) 8 (23.5) 8 (29.4) 6 (17.6) 10 (23.5)	0.439
Sauna	Everyday Few times per week Few times per month Rarely Never	0 (0) 0 (0) 0 (0) 7 (70) 3 (30)	0 (0) 0 (0) 2 (5.9) 11 (32.4) 21 (61.8)	0.476
Fast food	Everyday Few times per week Few times per month Rarely Never	0 (0) 1 (10) 3 (30) 6 (60) 0 (0)	0 (0) 6 (17.6) 12 (35.3) 11 (32.4) 5 (14.7)	1.389
Preservatives in food	Everyday Few times per week Few times per month Rarely Never	3 (30) 6 (60) 1 (10) 0 (0) 0 (0)	6 (17.6) 16 (47.1) 6 (17.6) 4 (11.8) 2 (5.9)	1.389
Laptop position	On the knees On the table Not using	2 (20) 5 (50) 3 (30)	0 (0) 30 (88.2) 4 (11.8)	2.500
Microwave	Everyday Few times per week Few times per month Rarely Never	0 (0) 2 (20) 3 (30) 5 (50) 0 (0)	3 (8.8) 10 (29.4) 5 (14.7) 3 (8.8) 13 (38.2)	4.044
Anabolic steroids	Yes No	0 (0) 10 (100)	0 (0) 34 (100)	1.533
Pharmaceuticals	Yes No	4 (40) 6 (60)	2 (5.9) 32 (94.1)	2.500
Physical activity	Everyday Few times per week Few times per month Rarely	1 (10) 2 (20) 4 (40) 3 (30)	5 (14.7) 8 (23.5) 5 (14.7) 16 (47.1)	3.75
Pesticides	Yes No	0 (0) 10 (100)	0 (0) 34 (100)	1.533
Smoking	Everyday Few times per week Few times per month Rarely Never	2 (20) 0 (0) 3 (30) 0 (0) 5 (50)	0 (0) 0 (0) 0 (0) 3 (8.8) 31 (91.2)	3.50
Alcohol consumption	Everyday Few times per week Few times per month Rarely Never	0 (0) 4 (40) 3 (30) 1 (10) 2 (20)	0 (0) 3 (8.8) 2 (5.9) 12 (35.3) 17 (50)	1.250
Stress level	Low Able to manage stress Level is alarming High	1 (10) 8 (80) 1 (10) 0 (0)	1 (2.9) 30 (88.2) 3 (8.8) 0 (0)	4.732

^1^ BMI: Body mass index.

**Table 6:** Risk factors and their association with male cases: *p*-values analysis.

Variable	Categories	Cases (n = 10), N%	Control (n = 34), N%	*p* Value
Age group	18–30 31–35 36–40 >41	3 (30) 6 (60) 1 (10) 0 (0)	14 (41.2) 10 (29.4) 8 (23.5) 2 (5.9)	4.732
BMI (kg/m^2^) ^1^	<18.5 18.5–25 25–30 >30	0 (0) 4 (40) 4 (40) 2 (20)	0 (0) 17 (50) 12 (35.3) 5 (14.7)	1.702
Education	Primary High school Higher school University University degree	0 (0) 1 (10) 0 (0) 0 (0) 9 (90)	1 (2.9) 9 (26.5 7 (20.6) 4 (11.8) 13 (38.2)	2.403
Sauna	Everyday Few times per week Few times per month Rarely Never	0 (0) 0 (0) 1 (10) 7 (70) 2 (20)	0 (0) 0 (0) 4 (11.8) 18 (52.3) 12 (35.3)	1.305
Fast food	Everyday Few times per week Few times per month Rarely Never	1 (10) 2 (20) 4 (40) 3 (30) 0 (0)	2 (5.9) 6 (17.6) 12 (17.6) 11 (32.4) 3 (8.8)	1.043
Preservatives in food	Everyday Few times per week Few times per month Rarely Never	3 (33.3) 5 (56.6) 1 (11.1) 0 (0) 0 (0)	9 (18.8) 19 (39.6) 10 (20.8) 6 (12.5) 4 (8.3)	1.204
Laptop position	On the knees On the table Not using	2 (20) 8 (80) 0 (0)	3 (8.8) 31 (91.2) 0 (0)	1.043
Microwave	Everyday Few times per week Few times per month Rarely Never	1 (11.1) 3 (33.3) 0 (0) 0 (0) 5 (55.6)	4 (11.8) 9 (26.5) 6 (17.6) 5 (14.7) 10 (29.4)	1.643
Anabolic steroids	Yes No	0 (0) 10 (100)	0 (0) 34 (100)	1.533
Pharmaceuticals	Yes No	3 (30) 7 (70)	2 (5.9) 32 (94.1)	1.435
Physical activity	Everyday Few times per week Few times per month Rarely	2 (20) 2 (20) 3 (30) 3 (30)	5 (10.4) 10 (20.8) 6 (17.6) 13 (38.2)	1.643
Pesticides	Yes No	0 (0) 10 (100)	0 (0) 34 (100)	1.533
Smoking	Everyday Few times per week Few times per month Rarely Never	3 (30) 0 (0) 0 (0) 1 (10) 6 (60)	8 (23.5) 3 (8.8) 1 (2.9) 2 (5.9) 20 (58.8)	1.653
Alcohol consumption	Everyday Few times per week Few times per month Rarely Never	0 (0) 1 (10) 6 (60) 2 (20) 1 (10)	7 (21.2) 2 (6.1) 2 (6.1) 0 (0) 22 (66.7)	1.534
Stress level	Low Able to manage stress Level is alarming High	0 (0) 8 (80) 2 (20) 0 (0)	1 (2.9) 31 (91.2) 2 (5.9) 0 (0)	1.632

^1^ BMI: Body mass index.

## Appendix A

**Table A1:** The description of female genes.

Gene Name	Loci	Inheritance	Phenotype
*MTHFR*—Methylenetetrahydrofolate Reductase	1p36.22	AR	Implantation, blood flow to the uterus, and fetal development
*FOLR1*—Folate Receptor Alpha	11q13.4	AR	Neurodegeneration due to cerebral folate transport deficiency
*SERPINE1*—endothelial plasminogen activator inhibitor-1 (PAI1)	7q22.1	AD, AR	Negatively regulates fibrinolysis and impairs the dissolution of clots
*TCN2*—Transcobalamin II	22q12.2	AD	Affects vitamin B12 transport
*CTH*—Cystathionine Gamma-Lyase	1p31.1	AR	Affects homocysteine metabolism, potentially impacting fertility due to altered folate levels and impaired embryo development
*SLC19A1*—Solute Carrier Family 19 Member 1	21q22.3	AR	Disrupts folate transport, affecting DNA synthesis
*NOS3*—Nitric Oxide Synthase 3	7q36.1	AR	Affects nitric oxide production, impacting ovarian function and vascular health
*CYP19A1*—Synaptonemal Complex Protein 3	Xq13	X-linked	Affects estrogen synthesis
*ESR1*—Estrogen Receptor 1	6q25.1	AD	Affects estrogen receptor function
*LIF*—Leukemia Inhibitory Factor	22q12.1	AR	Affects uterine receptivity, potentially leading to implantation failure
*HABP2*—Hyaluronan Binding Protein 2	10q26.13	AD	Impacts embryo implantation
*MLH3*—MutL Homolog 3	14q24.3	AR	Affects DNA repair
*TUBB8*—Tubulin Beta 8	10q24.32	AR	Impacts oocyte division
*ZP1*—Zona Pellucida Glycoprotein 1	11p11.2	AR	Affects oocyte fertilization
*PADI6*—Peptidyl Arginine Deiminase 6	1p36.13	AR	Affects oocyte quality
*TLE6*—Transducin-Like Enhancer of Split 6	9q21.2	AR	Impacts folliculogenesis
*F2*—Coagulation Factor II (Thrombin)	11p11.2	AR	Coagulation (thrombosis risk)
*CFTR*—Cystic Fibrosis Transmembrane Conductance Regulator	7q31.2	AR	Mucous viscosity
*CAPN10*—Calpain 10	2q37.3	AR	Insulin sensitivity
*AR*—Androgen Receptor	Xq11-12	X-linked recessive (AR)	Related to partial androgen insensitivity
*FSHR*—Follicle Stimulating Hormone Receptor	2q21	AD	Follicle development
*LHCGR*—Luteinizing Hormone/Chorionic Gonadotropin Receptor	2p21	AD	Ovarian hormone response
*ACE*—Angiotensin I Converting Enzyme	17q23.3	AD	Blood pressure regulation
*SOD2*—Superoxide Dismutase 2	6q25.3	AR	Oxidative stress regulation
*RNLS*—Renalase, FAD-Dependent Amine Oxidase	10q23.33	AD	Renalase enzyme
*VDR*—Vitamin D Receptor	12q13.11	AD	Vitamin D receptor function
*PALT2*—Patatin Like Phospholipase Domain Containing 2)	6p21.1	AR	Oocyte development

## Appendix B

**Table A2:** The description of male genes.

Gene Name	Loci	Inheritance	Phenotype
*CATSPER1*—cation channel sperm associated 2	11q13.1	AR	Asthenozoospermia
*POLG*—DNA Polymerase Gamma	15q25.1	AR/AD	Sperm quality and motility (linked to mitochondrial disorders).
*RPL23A*—Ribosomal Protein L23a	6p21.1	AD	Spermatogenesis
*RPL4*—Ribosomal Protein L4	15q22.2	AD	Spermatogenesis
*RPS27A*—Ribosomal Protein S27a	19q13.2	AD	Spermatogenesis
*RPS3*—Ribosomal Protein S3	9q34.11	AD	Spermatogenesis
*RPS8*—Ribosomal Protein S3Ribosomal Protein S8	1p34.2	AD	Spermatogenesis
*TOMM7*—Translocase of Outer Mitochondrial Membrane 7	19p13.2	AR	Spermatogenesis
*MTHFR*—Methylenetetrahydrofolate Reductase	1p36.22	AR	Affects sperm DNA integrity through DNA methylation, leading to an increased frequency of early spontaneous miscarriages
*APLF*—Aprataxin and PNKP Like Factor	4q35.1	AR	DNA damage repair
*CYB5R4*—Cytochrome B5 Reductase 4	1q21.1	AR	Preserves the cell from the buildup of reactive oxygen species (ROS)
*ERCC4*—ERCC Excision Repair 4, Endonuclease Catalytic Subunit	16p13.12	AR	Spermatogenesis, fertilization, and embryo development
*TNRFSF21*—Tumor Necrosis Factor Receptor Superfamily Member 21	19p13.2	AR	Promotes apoptosis, mediated by BAX, involved in the mitochondrial apoptotic process
*MORC1*—MORC Family CW-Type Zinc Finger 1	3q13.32	AR	Participates in apoptosis, involved in early spermatogenesis
*PIWIL1*—Piwi Like RNA-Mediated Gene Silencing 1	12q24.33	AR	Spontaneous regeneration of stem cells, inhibition of DNA replication
*ZFAND6*—Zinc Finger AN1-Type Containing 6	2q37.3	AR	Regulates TNF alpha-induced NF kappa-B activation and apoptosis
*DPY19L2*—Dpy-19 Like 2	12q14.2	AR	Expressed in the testes and is required for elongation of the sperm head and acrosome formation during spermatogenesis
*ADAM3A*—Metallopeptidase domain 3A	Xq11.2	AD	Participates in gamete fusion during fertilization
*NXF2*—nuclear RNA export factor 2	Xq22.1	-	Spermatogenesis
*SIRPB1*—Signal-Regulatory Protein Beta 1	20p13	AD	Spermatogenesis
*FSHR*—Follicle Stimulating Hormone Receptor	2p21	AD	Luteinizing hormone resistance and Leydig cell hypoplasia
*LHCGR*—Luteinizing Hormone/Choriogonadotropin Receptor	2p16.3	AD	Testotoxicosis, hypogonadotropic hypogonadism, Leydig cell adenoma with precocious puberty, and male pseudohermaphrod itism with Leydig cell hypoplasia
*AR*—Androgen Receptor	Xq11-12	X-linked recessive (AR)	Related to partial androgen insensitivity

## References

[1] Patel B., Parets S., Akana M., Kellogg G., Jansen M., Chang C., Cai Y., Fox R., Niknazar M., Shraga R. (2018). Comprehensive genetic testing for female and male infertility using next-generation sequencing. J. Assist. Reprod. Genet..

[2] Harper J.C., Aittomäki K., Borry P., Cornel M.C., de Wert G., Dondorp W., Geraedts J., Gianaroli L., Ketterson K., Liebaers I. (2017). Recent developments in genetics and medically assisted reproduction: From research to clinical applications. Eur. J. Hum. Genet..

[3] Romualdi D., Ata B., Bhattacharya S., Bosch E., Costello M., Gersak K., Homburg R., Mincheva M., Norman R.J., Piltonen T. (2023). Evidence-based guideline: Unexplained infertility. Hum. Reprod..

[4] Altmäe S., Stavreus-Evers A., Ruiz J.R., Laanpere M., Syvänen T., Yngve A., Salumets A., Nilsson T.K. (2010). Variations in folate pathway genes are associated with unexplained female infertility. Fertil. Steril..

[5] Nishiyama S., Kishi T., Kato T., Suzuki M., Bolor H., Nishizawa H., Iwata N., Udagawa Y., Kurahashi H. (2010). A rare synaptonemal complex protein 3 gene variant in unexplained female infertility. Mol. Hum. Reprod..

[6] Zorrilla M., Yatsenko A.N. (2013). The Genetics of Infertility: Current Status of the Field. Curr. Genet. Med. Rep..

[7] Karatas A., Eroz R., Bahadır A., Keskin F., Ozlu T., Ozyalvaclı M.E. (2014). Endothelial Nitric Oxide Synthase Gene Polymorphisms (Promoter -786T/C, Exon 894 G/T and Intron G10T) in Unexplained Female Infertility. Gynecol. Obstet. Investig..

[8] Altmäe S., Hallerc K., Peters M., Saare M., Hovatta O., Stavreus-Evers A., Velthut A., Karro H., Metspalu A., Salumets A. (2009). Aromatase gene (CYP19A1) variants, female infertility and ovarian stimulation outcome: A preliminary report. Reprod. BioMedicine Online.

[9] Ayvaz Ö.Ü., Ekmekçi A., Baltaci V., Onen H.I., Unsal E. (2009). Evaluation of in vitro fertilization parameters and estrogen receptor alpha gene poly-morphisms for women with unexplained infertility. J. Assist. Reprod. Genet..

[10] Steck T., Giess R., Suetterlin M.W., Bolland M., Wiest S., Poehls U.G., Dietl J. (2004). Leukaemia inhibitory factor (LIF) gene mutations in women with unexplained infertility and re-current failure of implantation after IVF and embryo transfer. Eur. J. Obstet. Gynecol. Reprod. Biol..

[11] Novotný Z., Křížan J., Šíma R., Šíma P., Uher P., Zech N., Hüttelová R., Baborová P., Ulčová-Gallová Z., Šubrt I. (2009). Leukaemia Inhibitory Factor (LIF) Gene Mutations in Women Diagnosed with Unexplained Infertility and Endometriosis Have a Negative Impact on the IVF Outcome a Pilot Study. Folia Biol..

[12] Du J.-W., Tao X.-R., Xu K.-Y., Fang L.-Y., Qi X.-L. (2011). Polymorphisms in estrogen receptor-α are associated with idiopathic female infertility. Mol. Med. Rep..

[13] Coulam C.B., Jeyendran R. (2009). Thrombophilic gene polymorphisms are risk factors for unexplained infertility. Fertil. Steril..

[14] Altamae S., Kunovac Kallac T., Fridén B., Stavreus-Evers A. (2010). Variation in Hyaluronan-Binding Protein 2 (HABP2) Promoter Region is Associated with Unexplained Female Infertility. Reprod. Sci..

[15] Fatinia C., Contib L., Turillazzi V., Sticchi E., Romagnuolo I., Milanini M.N., Cozzi C., Abbate R., Noci I. (2012). Unexplained infertility: Association with inherited thrombophilia. Thromb. Res..

[16] Pashaiefar H., Sheikhha M.H., Kalantar S.M., Jahaninejad T., Zaimy M.A., Ghasemi N. (2013). Analysis of MLH3 C2531T polymorphism in Iranian women with unexplained infertility. Iran. J. Reprod. Med..

[17] Christofolini D.M., Cavalheiro C.M., Teles J.S., Lerner T.G., Brandes A., Bianco B., Barbosa C.P. (2011). Promoter -817C>T Variant of B Lymphocyte Stimulator Gene (BLyS) and Susceptibility to Endometriosis-Related Infertility and Idiopathic Infertility in Brazilian Population. Scand. J. Immunol..

[18] Laissue P. (2015). Aetiological coding sequence variants in non-syndromic premature ovarian failure: From genetic linkage analysis to next generation sequencing. Mol. Cell. Endocrinol..

[19] Lorenzi D., Fernández C., Bilinski M., Fabbro M., Galain M., Menazzi S., Miguens M., Perassi P.N., Fulco M.F., Kopelman S. (2020). First custom next-generation sequencing infertility panel in Latin America: Design and first results. JBRA Assist. Reprod..

[20] Xu Y., Shi Y., Fu J., Yu M., Feng R., Sang Q., Liang B., Chen B., Qu R., Li B. (2016). Mutations in PADI6 Cause Female Infertility Characterized by Early Embryonic Arrest. Am. J. Hum. Genet..

[21] Hart R.J. (2016). Physiological Aspects of Female Fertility: Role of the Environment, Modern Lifestyle, and Genetics. Physiol. Rev..

[22] Al-Mutawa J. (2016). Interaction with angiotensin-converting enzyme-encoding gene in female infertility: Insertion and deletion polymorphism studies. Saudi J. Biol. Sci..

[23] Kydonopoulou K., Delkos D., Rousso D., Ilonidis G., Mandala E. (2017). Association of plasminogen activator inhibitor-type 1 (PAI-1) -675 4G/5G polymorphism with unexplained female infertility. Hippokratia.

[24] Das V., Misra D., Agrawal S., Agrawal A., Pandey A. (2015). Hyperhomocysteinemia and MTHFR gene 677 C>T polymorphism: Questionable role in female infertility. Int. J. Reprod. Contracept. Obstet. Gynecol..

[25] Geisinger A., Benavente R. (2016). Mutations in Genes Coding for Synaptonemal Complex Proteins and Their Impact on Human Fertility. Cytogenet. Genome Res..

[26] Mohtaram S., Sheikhha M.H., Honarvar N., Sazegari A., Maraghechi N., Feizollahi Z., Ghasemi N. (2016). An Association Study of the *SLC19A1* Gene Polymorphisms/Haplotypes with Idiopathic Recurrent Pregnancy Loss in an Iranian Population. Genet. Test. Mol. Biomarkers.

[27] Liaqat S., Hasnain S., Muzammil S., Hayat S. (2015). Polymorphism analysis in estrogen receptors alpha and beta genes and their association with infertile population in Pakistan. EXCLI J..

[28] Cao Y., Zhang Z., Zheng Y., Yuan W., Wang J., Liang H., Chen J., Du J., Shen Y. (2014). The association of idiopathic recurrent early pregnancy loss with polymorphisms in folic acid metabolism-related genes. Genes Nutr..

[29] Dorostghoal M., Ghaffari H.-O., Moramezi F., Keikhah N. (2018). Overexpression of Endometrial Estrogen Receptor-Alpha in The Window of Implantation in Women with Unexplained Infertility. Int. J. Fertil. Steril..

[30] Pournouralia M., Tarangb A., Haghighi S.F., Yousefi M., Bahadori M.H. (2016). Polymorphism variant of MnSOD A16V and risk of female infertility in northern Iran. Taiwan. J. Obstet. Gynecol..

[31] Fatima S.S., Rehman R., Martins R.S., Alam F., Ashraf M. (2019). Single nucleotide polymorphisms in Renalase and KCNQ1 genes and female infertility: A cross-sectional study in Pakistan. Andrologia.

[32] Djurovic J., Stamenkovic G., Todorovic J., Aleksic N., Stojkovic O. (2018). Polymorphisms and haplotypes in VDR gene are associated with female idiopathic in-fertility. J. Hum. Fertil..

[33] Huang W., Wang J., Pang M., Zhao Q., Kong L., Mao Y., Li W., Liang Β. (2019). Copy number variations in female infertility in China. Balk. J. Med. Genet..

[34] Podarab T.L., Kaart T., Peters M., Salumets A. (2015). Genetic variants associated with female reproductive ageing—Potential markers for assessing ovarian function and ovarian stimulation outcome. Reprod. Biomed. Online.

[35] Wu L., Chen H., Li D., Song D., Chen B., Yan Z., Lyu Q., Wang L., Kuang Y., Li B. (2019). Novel mutations in PATL2: Expanding the mutational spectrum and corresponding phenotypic var-iability associated with female infertility. J. Hum. Genet..

[36] Laanperea M., Altmaebc S., Kaart T., Stavreus-Evers A., Nilsson T.K., Salumets A. (2011). Folate-metabolizing gene variants and pregnancy outcome of IVF. Reprod. Biomed. Online.

[37] Park H.S., Kim J.O., An H.J., Ryu C.S., Ko E.J., Kim Y.R., Ahn E.H., Lee W.S., Kim J.H., Kim N.K. (2019). Genetic polymorphisms of the cobalamin transport system are associated with idiopathic recurrent implantation failure. J. Assist. Reprod. Genet..

[38] Najafia T., Novin M.G., Ghazi R., Khorram O. (2012). Altered endometrial expression of endothelial nitric oxide synthase in women with unex-plained recurrent miscarriage and infertility. Reprod. BioMedicine Online.

[39] Wu M., Yin Y., Zhao M., Hu L., Chen Q. (2013). The low expression of leukemia inhibitory factor in endometrium: Possible relevant to unexplained infertility with multiple implantation failures. Cytokine.

[40] Aghajanova L., Altmäe S., Bjuresten K., Hovatta O., Landgren B.-M., Stavreus-Evers A. (2009). Disturbances in the LIF pathway in the endometrium among women with unexplained infertility. Fertil. Steril..

[41] Vagnini L.D., Renzi A., Petersen B., Canas M.D.C.T., Petersen C.G., Mauri A.L., Mattila M.C., Ricci J., Dieamant F., Oliveira J.B.A. (2019). Association between estrogen receptor 1 (ESR1) and leukemia inhibitory factor (LIF) polymorphisms can help in the prediction of recurrent implantation failure. Fertil. Steril..

[42] Margioula-Siarkou C., Prapas Y., Petousis S., Milias S., Ravanos K., Dagklis T., Kalogiannidis I., Mavromatidis G., Haitoglou C., Prapas N. (2017). LIF endometrial expression is impaired in women with unexplained infertility while LIF-R expression in all infertility sub-groups. Cytokine.

[43] Hu M., Li S., Wang N., Wu Y., Jin F. (2016). Impact of DNA mismatch repair system alterations on human fertility and related treatments. J. Zhejiang Univ.-Sci. B.

[44] Chen B., Wang W., Peng X., Jiang H., Zhang S., Li D., Li B., Fu J., Kuang Y., Sun X. (2018). The comprehensive mutational and phenotypic spectrum of TUBB8 in female infertility. Eur. J. Hum. Genet..

[45] Wang A.-C., Zhang Y.-S., Wang B.-S., Zhao X.-Y., Wu F.-X., Zhai X.-H., Sun J.-X., Mei S.-Y. (2018). Mutation analysis of theTUBB8 gene in primary infertile women with arrest in oocyte maturation. Gynecol. Endocrinol..

[46] Zhou Z., Ni C., Wu L., Chen B., Xu Y., Zhang Z., Mu J., Li B., Yan Z., Fu J. (2019). Novel mutations in ZP1, ZP2, and ZP3 cause female infertility due to abnormal zona pellucida formation. Qual. Life Res..

[47] Alazami A.M., Awad S.M., Coskun S., Al-Hassan S., Hijazi H., Abdulwahab F.M., Poizat C., Alkuraya F.S. (2015). TLE6 mutation causes the earliest known human embryonic lethality. Genome Biol..

[48] Foresta C., Ferlin A., Gianaroli L., Dallapiccola B. (2002). Guidelines for the appropriate use of genetic tests in infertile couples. Eur. J. Hum. Genet..

[49] Corbo R.M., Ulizzi L., Piombo L., Scacchi R. (2010). Association of ACE I/D polymorphism and recurrent miscarriages in an Italian population with a pre-modern reproductive pattern. Ann. Hum. Biol..

[50] Ye Y., Vattai A., Zhang X., Zhu J., Thaler C.J., Mahner S., Jeschke U., von Schönfeldt V. (2017). Role of Plasminogen Activator Inhibitor Type 1 in Pathologies of Female Reproductive Diseases. Int. J. Mol. Sci..

[51] Buchholz T., Lohse P., Rogenhofer N., Kosian E., Pihusch R., Thaler C.J. (2003). Polymorphisms in the ACE and PAI-1 genes are associated with recurrent spontaneous miscar-riages. Hum. Reprod..

[52] Subrt I., Ulcova-Gallova Z., Cerna M., Hejnalova M., Slovanova J., Bibkova K., Micanova Z. (2013). Recurrent Pregnancy Loss, Plasminogen Activator Inhibitor-1 (-675) 4G/5G Polymorphism and Antiphospholipid Antibodies in Czech Women. Am. J. Reprod. Immunol..

[53] Reginatto M.W., Pizarro B.M., Antunes R.A., Mancebo A.C.A., Hoffmann L., Fernandes P., Areas P., Chiamolera M.I., Silva R., de Souza M.D.C.B. (2018). Vitamin D Receptor TaqI Polymorphism Is Associated with Reduced Follicle Number in Women Utilizing Assisted Reproductive Technologies. Front. Endocrinol..

[54] Huang L., Tong X., Wang F., Luo L., Jin R., Fu Y., Zhou G., Li D., Song G., Liu Y. (2018). Novel mutations in PATL2 cause female infertility with oocyte germinal vesicle arrest. Hum. Reprod..

[55] Chen B., Zhang Z., Sun X., Kuang Y., Mao X., Wang X., Yan Z., Li B., Xu Y., Yu M. (2017). Biallelic Mutations in PATL2 Cause Female Infertility Characterized by Oocyte Maturation Arrest. Am. J. Hum. Genet..

[56] Maddirevula S., Coskun S., Alhassan S., Elnour A., Alsaif H.S., Ibrahim N., Abdulwahab F., Arold S.T., Alkuraya F.S. (2017). Female Infertility Caused by Mutations in the Oocyte-Specific Translational Repressor PATL2. Am. J. Hum. Genet..

[57] Christou-Kent M., Kherraf Z., Amiri-Yekta A., Le Blévec E., Karaouzène T., Conne B., Escoffier J., Assou S., Guttin A., Lambert E. (2018). PATL 2 is a key actor of oocyte maturation whose invalidation causes infertility in women and mice. EMBO Mol. Med..

[58] Esteves S. (2013). A clinical appraisal of the genetic basis in unexplained male infertility. J. Hum. Reprod. Sci..

[59] Lishko P.V., Kirichok Y. (2010). The role of Hv1 and CatSper channels in sperm activation. J. Physiol..

[60] Carlson A.E., Burnett L.A., del Camino D., Quill T.A., Hille B., Chong J.A., Moran M.M., Babcock D.F. (2009). Pharmacological Targeting of Native CatSper Channels Reveals a Required Role in Maintenance of Sperm Hyperactivation. PLoS ONE.

[61] Rovio A.T., Marchington D.R., Donat S., Schuppe H.-C., Abel J., Fritsche E., Elliott D.J., Laippala P., Ahola A.L., McNay D. (2001). Mutations at the mitochondrial DNA polymerase (POLG) locus associated with male infertility. Nat. Genet..

[62] Jensen M., Leffers H., Petersen J.H., Andersen A.N., Jørgensen N., Carlsen E., Jensen T.K., Skakkebæk N.E., Meyts E.R. (2004). Frequent polymorphism of the mitochondrial DNA polymerase gamma gene (POLG) in patients with normal spermiograms and unexplained subfertility. Hum. Reprod..

[63] Bonache S., Mata A., Ramos M.D., Bassas L., Larriba S. (2012). Sperm gene expression profile is related to pregnancy rate after insemination and is predictive of low fecundity in normozoospermic men. Hum. Reprod..

[64] Tamburrino L., Marchiani S., Vicini E., Muciaccia B., Cambi M., Pellegrini S., Forti G., Muratori M., Baldi E. (2015). Quantification of CatSper1 expression in human spermatozoa and relation to functional parameters. Hum. Reprod..

[65] Oviedo N., Ortiz-Borrayo L., Hernández-Sánchez J., Jiménez-Badillo S.E., Tesoro-Cruz E., Moreno-Navor E., Aguirre-Alvarado C., Bekker-Méndez V.C. (2018). Human CATSPER1 Promoter Is Regulated by CREB1 and CREMτ Transcriptional Factors In Vitro. Arch. Med. Res..

[66] Li H.-G., Liao A.-H., Ding X.-F., Zhou H., Xiong C.-L. (2006). The expression and significance of CATSPER1 in human testis and ejaculated spermatozoa. Asian J. Androl..

[67] Hildebrand M.S., Avenarius M.R., Fellous M., Zhang Y., Meyer N.C., Auer J., Serres C., Kahrizi K., Najmabadi H., Beckmann J.S. (2010). Genetic male infertility and mutation of CATSPER ion channels. Eur. J. Hum. Genet..

[68] Demain L.A.M., Conway G.S., Newman W.G. (2016). Genetics of mitochondrial dysfunction and infertility. Clin. Genet..

[69] Poongothai J. (2013). Mitochondrial DNA polymerase gamma gene polymorphism is not associated with male infertility. J. Assist. Reprod. Genet..

[70] Ferlin A., Foresta C. (2014). New genetic markers for male infertility. Curr. Opin. Obstet. Gynecol..

[71] Bartolomé S.L., Arnau L.B. (2014). In Vitro Method for Predicting Semen Fertility. U.S. Patent.

[72] Gong M., Dong W., He T., Shi Z., Huang G., Ren R., Huang S., Qiu S., Yuan R. (2015). MTHFR 677C>T Polymorphism Increases the Male Infertility Risk: A Meta-Analysis Involving 26 Studies. PLoS ONE.

[73] Park J.H., Lee H.C., Jeong Y.-M., Chung T.-G., Kim H.-J., Kim N.K., Lee S.-H., Lee S. (2005). MTHFR C677T polymorphism associates with unexplained infertile male factors. J. Assist. Reprod. Genet..

[74] Liu K., Zhao R., Shen M., Ye J., Li X., Huang Y., Hua L., Wang Z., Li J. (2015). Role of genetic mutations in folate-related enzyme genes on Male Infertility. Sci. Rep..

[75] Suman L., Yu-Mi J., Lee S.-H., Cha K.Y., Chung T.-G., Yoon T.-K. (2003). The C677T polymorphism in methylenetetrahydrofolate reductase (MTHFR) gene associates with unexplained male infertility with severe OAT. Fertil. Steril..

[76] Irfan M., Ismail M., Beg M.A., Shabbir A., Kayani A.R., Raja G.K. (2016). Association of the *MTHFR* C677T (rs1801133) polymorphism with idiopathic male infertility in a local Pakistani population. Balk. J. Med. Genet..

[77] Karimian M., Colagar A.H. (2016). Association of C677T transition of the human methylenetetrahydrofolate reductase (MTHFR) gene with male infertility. Reprod. Fertil. Dev..

[78] Najafipour R., Moghbelinejad S., Aleyasin A., Jalilvand A. (2017). Effect of B9 and B12 vitamin intake on semen parameters and fertility of men with MTHFR polymorphisms. Andrology.

[79] Liu Y., Zhang F., Dai L. (2019). C677T poly-morphism increases the risk of early spontaneous abortion. J. Assist. Reprod. Genet..

[80] Ullah N., Mansoor A., Micheal S., Mirza B., Qamar R., Mazhar K., Siddiqi S. (2018). *MTHFR* polymorphisms as Risk for Male Infertility in Pakistan and its Comparison with Socioeconomic Status in the World. Pers. Med..

[81] Poorang S., Abdollahi S., Anvar Z., Tabei S., Jahromi B.-N., Moein-Vaziri N., Gharesi-Fard B., Banaei M., Dastgheib S. (2018). The Impact of Methylenetetrahydrofolate Reductase (MTHFR) Sperm Methylation and Variants on Semen Parameters and the Chance of Recurrent Pregnancy Loss in the Couple. Clin. Lab..

[82] Schilit S.L., Menon S., Friedrich C., Kammin T., Wilch E., Hanscom C., Jiang S., Kliesch S., Talkowski M.E., Tüttelmann F. (2019). SYCP2 Translocation-Mediated Dysregulation and Frameshift Variants Cause Human Male Infertility. Am. J. Hum. Genet..

[83] Pereira N., Cheung S., Parrella A., O’Neill C., Nikprelevic N., Rosenwaks Z., Palermo G. (2017). Investigating the role of sperm-specific RNA to screen men with unexplained infertility. Fertil. Steril..

[84] Cheung S., Parrella A., Rosenwaks Z., Palermo G.D. (2019). Genetic and epigenetic profiling of the infertile male. PLoS ONE.

[85] Razavi S.M., Sabbaghian M., Jalili M., Divsalar A., Wolkenhauer O., Salehzadeh-Yazdi A. (2017). Comprehensive functional enrichment analysis of male infertility. Sci. Rep..

[86] Boissonnas C.C., Jouannet P., Jammes H. (2013). Epigenetic disorders and male subfertility. Fertil. Steril..

[87] Hamada A., Esteves S.C., Nizza M., Agarwal A. (2012). Unexplained Male infertility: Diagnosis and Management. Int. Braz. J. Urol..

[88] Sharma A. (2017). Male Infertility; Evidences, Risk Factors, Causes, Diagnosis and Management in Human. Ann. Clin. Lab. Res..

[89] Shah K., Sivapalan G., Gibbons N., Tempest H., Griffin D.K. (2003). The genetic basis of infertility. Reproduction.

[90] Cariati F., D’argenio V., Tomaiuolo R. (2019). The evolving role of genetic tests in reproductive medicine. J. Transl. Med..

[91] Hempfling A.L., Lim S.L., Adelson D.L., Evans J., O’connor A.E., Qu Z.P., Kliesch S., Weidner W., O’bryan M.K., Bergmann M. (2017). Expression patterns of HENMT1 and PIWIL1 in human testis: Implications for transposon expression. Reproduction.

[92] Friemel C., Ammerpohl O., Gutwein J., Schmutzler A.G., Caliebe A., Kautza M., von Otte S., Siebert R., Bens S. (2014). Array-based DNA methylation profiling in male infertility reveals allele-specific DNA methylation in PIWIL1 and PIWIL2. Fertil. Steril..

[93] Gu A., Ji G., Shi X., Long Y., Xia Y., Song L., Wang S., Wang X. (2010). Genetic variants in Piwi-interacting RNA pathway genes confer susceptibility to spermatogenic failure in a Chinese population. Hum. Reprod..

[94] Gou L.-T., Kang J.-Y., Dai P., Wang X., Li F., Zhao S., Zhang M., Hua M.-M., Lu Y., Zhu Y. (2017). Ubiquitination-Deficient Mutations in Human Piwi Cause Male Infertility by Impairing Histone-to-Protamine Exchange during Spermiogenesis. Cell.

[95] Gu A., Ji G., Zhou Y., Long Y., Shi X., Fu G., Wang S., Song L., Wang X. (2010). Polymorphisms of nucleotide-excision repair genes may contribute to sperm DNA fragmentation and male infertility. Reprod. Biomed. Online.

[96] Cao Y.-X., Li W., Shang Y.-L., Zhu F.-X., Yan J., Chen L., Tang W.-H., Xiao S., Mo W.-K., Zhang Z.-G. (2019). Novel DPY19L2 variants in globozoospermic patients and the overcoming this male infertility. Asian J. Androl..

[97] Ray P.F., Toure A., Metzler-Guillemain C., Mitchell M.J., Arnoult C., Coutton C. (2016). Genetic abnormalities leading to qualitative defects of sperm morphology or function. Clin. Genet..

[98] Krzastek S.C., Smith R.P., Kovac J.R. (2020). Future diagnostics in male infertility: Genomics, epigenetics, metabolomics and proteomics. Transl. Androl. Urol..

[99] Sivasankaran R., Bassouvalingam K., Ilangovan R., Yuvaraj S., Sridhar M., Venkataraman P., Srinivasan N., Aruldhas M.M. (2007). Modulation of antioxidant defense system by the environmental fungicide car-bendazim in Leydig cells of rats. Reprod. Toxicol..

[100] Nguyen R.H., Wilcox A.J., Skjaerven R., Baird D.D., Skjærven R. (2007). Men’s body mass index and infertility. Hum. Reprod..

[101] Prusakiewicz J.J., Harville H.M., Zhang Y., Ackermann C., Voorman R.L. (2007). Parabens inhibit human skin estrogen sulfotransferase activity: Possible link to paraben estrogenic effects. Toxicology.

[102] Ahmed T.M.K., Bakr M.M. (2022). Side Effects of Preservatives on Human Life. Sci. Res. J. Pharm..

[103] McGrady A.V. (1984). Effects of Psychological Stress on Male Reproduction: A Review. J. Reprod. Syst..

[104] Nargund V.H. (2015). Effects of psychological stress on male fertility. Nat. Rev. Urol..

[105] Bala R., Singh V. (2021). Environment, Lifestyle, and Female Infertility. Reprod. Sci..

